# Crystal structure of (pyridin-2-yl­methyl­idene)(tri­phenyl­meth­yl)amine

**DOI:** 10.1107/S160053681401959X

**Published:** 2014-09-06

**Authors:** Chatphorn Theppitak, Manlika Meesangkaew, Songwuit Chanthee, Nimit Sriprang, Kittipong Chainok

**Affiliations:** a Department of Chemistry, Faculty of Science, Naresuan University, Mueang, Phitsanulok 65000, Thailand; b Department of Physics, Faculty of Science and Technology, Thammasat University, Khlong Luang, Pathum Thani 12120, Thailand

**Keywords:** crystal structure, amine, pyridin-2-yl­methyl­idene, trit­yl, Schiff base ligands, magnetism

## Abstract

The title Schiff base compound, C_25_H_20_N_2_, crystallizes with two independent mol­ecules (*A* and *B*) in the asymmetric unit. In both mol­ecules, the imine group is approximately coplanar with the pyridine ring, with N—C—C—N torsion angles of 170.1 (3) and −172.0 (3) Å. In the crystal, *A* and *B* dimers are linked by pairs of C—H⋯π inter­actions and further C—H⋯π bonds link the dimers into a three-dimensional network.

## Related literature   

For the use of the pyridin-2-ylmethanimine Schiff base ligands for the development of a new generation of memory devices, multifunctional materials, and other magnetic applications, see: Capes *et al.* (2000 ▶); Guionneau *et al.* (2001 ▶); Létard *et al.* (1997 ▶, 1998 ▶); Liu *et al.* (2010 ▶); Murray (2008 ▶); Goodwin (2004 ▶); Gupta & Sutar (2008 ▶). For van der Waals radii, see: Bondi (1964 ▶)
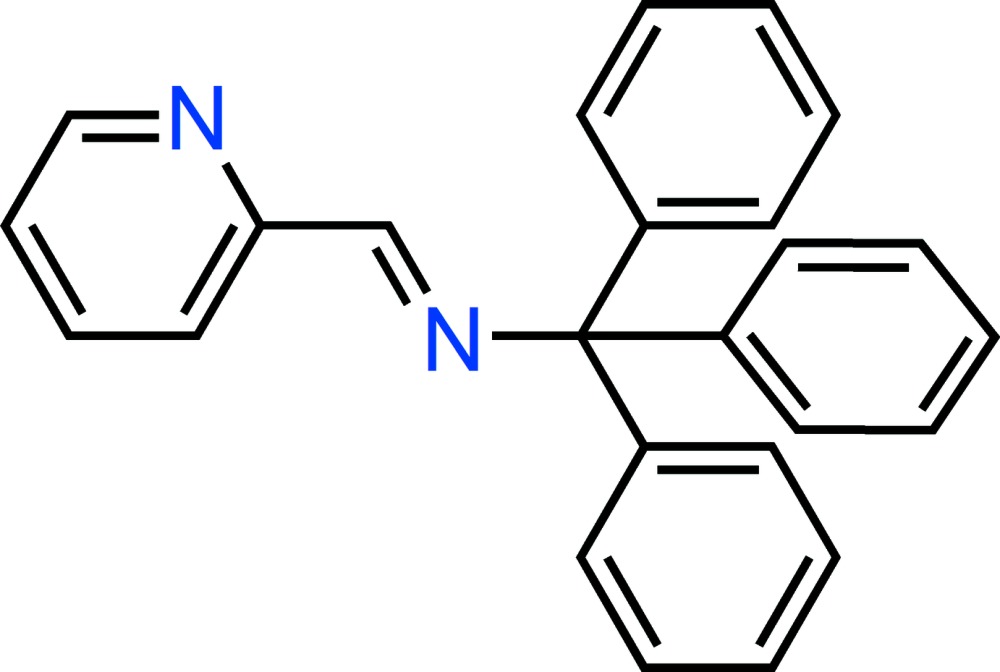


## Experimental   

### Crystal data  

C_25_H_20_N_2_
*M* *_r_* = 348.43
Orthorhombic, 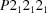

*a* = 7.0719 (5) Å
*b* = 16.3972 (8) Å
*c* = 31.880 (2) Å
*V* = 3696.7 (4) Å^3^
*Z* = 8
Cu *K*α radiation
μ = 0.56 mm^−1^
*T* = 173 K
0.30 × 0.26 × 0.22 mm

### Data collection  

Agilent Xcalibur (Sapphire3, Gemini ultra) diffractometer
Absorption correction: multi-scan (*CrysAlis PRO* and *CrysAlis RED*; Agilent, 2012 ▶) *T*_min_ = 0.849, *T*_max_ = 0.886
21735 measured reflections
6965 independent reflections
5059 reflections with *I* > 2σ(*I*)
*R*_int_ = 0.058

### Refinement  

*R*[*F* ^2^ > 2σ(*F* ^2^)] = 0.057
*wR*(*F* ^2^) = 0.153
*S* = 1.00
6965 reflections
487 parameters
H-atom parameters constrained
Δρ_max_ = 0.36 e Å^−3^
Δρ_min_ = −0.18 e Å^−3^

### 

Data collection: *CrysAlis PRO* (Agilent, 2012 ▶); cell refinement: *CrysAlis PRO*; data reduction: *CrysAlis PRO*; program(s) used to solve structure: *SHELXS97* (Sheldrick, 2008 ▶); program(s) used to refine structure: *SHELXL97* (Sheldrick, 2008 ▶); molecular graphics: *ORTEP-3 for Windows* (Farrugia, 2012 ▶) and *DIAMOND* (Brandenburg, 2006 ▶); software used to prepare material for publication: *publCIF* (Westrip, 2010 ▶).

## Supplementary Material

Crystal structure: contains datablock(s) global, I. DOI: 10.1107/S160053681401959X/tk5243sup1.cif

Structure factors: contains datablock(s) I. DOI: 10.1107/S160053681401959X/tk5243Isup2.hkl

Supporting information file. DOI: 10.1107/S160053681401959X/tk5243Isup3.cdx

Supporting information file. DOI: 10.1107/S160053681401959X/tk5243Isup4.cml

**Fig. 1.**
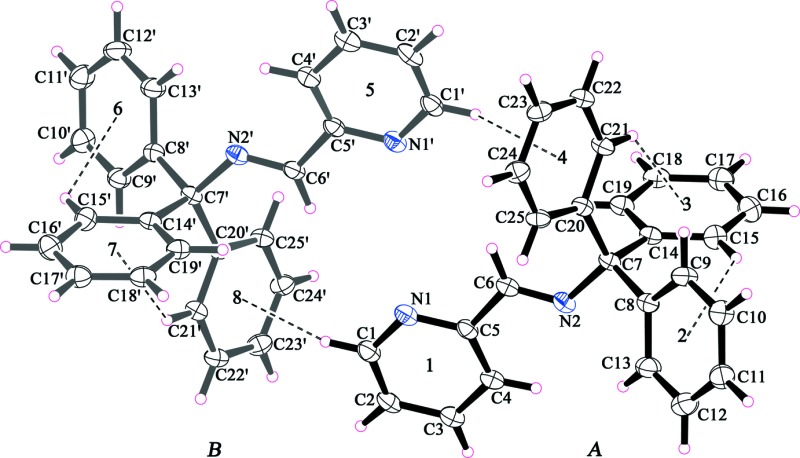
A view of the two symmetry-independent molecules (A and B) in (I), showing the atom-numbering scheme. Displacement ellipsoids are drawn at the 30% probability level. Covalent bonds in A and B molecules are shaded differently. The labeling scheme, 1–8, applied to the aromatic rings are used to identify the rings in the subsequent discussion.

A B A B . DOI: 10.1107/S160053681401959X/tk5243fig1.tif
A view of the two symmetry-independent mol­ecules (*A* and *B*) in (I), showing the atom-numbering scheme. Displacement ellipsoids are drawn at the 30% probability level. Covalent bonds in *A* and *B* mol­ecules are shaded differently. The labeling scheme, 1–8, applied to the aromatic rings are used to identify the rings in the subsequent discussion.

**Fig. 2.**
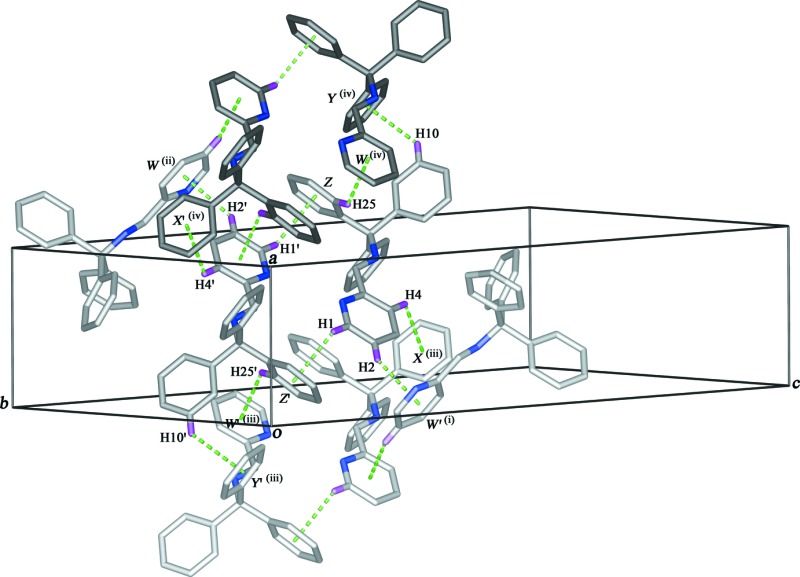
A packing diagram for (I), displaying the intermolecular ef C—H···π interactions (dashed lines). For clarity, only H atoms involved in C—H···π hydrogen bonding have been included. [Symmetry codes: (i) 1–x, 1/2 + y, 3/2–z; (ii) –x, y–1/2, 3/2–z; (iii) 1 + x, y, z; (iv) x–1, y, z].

Click here for additional data file.ef y y x y z x y z . DOI: 10.1107/S160053681401959X/tk5243fig2.tif
A packing diagram for (I), displaying the inter­molecular *ef* C—H⋯π inter­actions (dashed lines). For clarity, only H atoms involved in C—H⋯π hydrogen bonding have been included. [Symmetry codes: (i) 1–x, 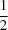 + *y*, 3/2–z; (ii) –x, *y*–1/2, 3/2–z; (iii) 1 + *x*, *y*, *z*; (iv) *x*–1, *y*, *z*].

CCDC reference: 1021733

Additional supporting information:  crystallographic information; 3D view; checkCIF report

## Figures and Tables

**Table 1:** Hydrogen-bond geometry (Å, °) *Cg*1 is the centroid of the N1, C1–C5 ring, *Cg*2 of ring C8–C13, *Cg*3 of ring C14–C19, *Cg*4 of ring C20–C25, *Cg*5 of ring N1′, C1′–C5′, *Cg*6 of ring C8′–C13′, *Cg*7 of ring C14′–C19′ and *Cg*8 of ring C20′–C25′.

D—H⋯A	D—H	H⋯A	H⋯Plane	D⋯A	D—H⋯A
C1—H1⋯*Cg*8	0.95	2.819	2.786 (3)	3.573	137
C1′—H1′⋯*Cg*4	0.95	2.789	2.749 (3)	3.545	137
C2—H2⋯*Cg*5^i^	0.95	2.769	2.751 (3)	3.591	145
C2′—H2′⋯*Cg*1^ii^	0.95	2.789	2.717 (3)	3.555	138
C4—H4⋯*Cg*2^iii^	0.95	3.150	2.982 (3)	3.692	118
C4′—H4′⋯*Cg*6^iv^	0.95	3.169	2.942 (3)	3.687	116
C9—H9⋯*Cg*4	0.95	3.104	2.533 (4)	3.670	120
C9′—H9′⋯*Cg*8	0.95	3.061	2.526 (4)	3.642	121
C10—H10⋯*Cg*3^iv^	0.95	3.077	3.038 (4)	3.823	136
C10′—H10′⋯*Cg*7^iii^	0.95	3.133	3.067 (4)	3.871	136
C15—H15⋯*Cg*2	0.95	3.030	2.386 (4)	3.717	130
C15′—15′⋯*Cg*6	0.95	3.027	2.387 (4)	3.715	131
C21—H21⋯*Cg*3	0.95	2.897	2.424 (4)	3.614	133
C21′—H21′⋯*Cg*7	0.95	2.886	2.435 (4)	3.604	133
C25—H25⋯*Cg*1^iv^	0.95	2.859	2.824 (3)	3.493	125
C25′—H25′⋯*Cg*5^iii^	0.95	2.865	2.824 (3)	3.496	125

Symmetry codes: (i) 1 − *x*, 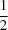 + *y*, 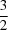 − *z*; (ii) −*x*, *y* − 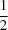, 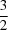 − *z*; (iii) 1 + *x*, *y*, *z*; (iv) *x* − 1, *y*, *z*.

## supplementary crystallographic information

### S1. Comment

Schiff bases and their complexes have received intense attention owing to their structural and functional diversities (Gupta & Sutar, 2008; Liu *et al.*, 2010). Much effort has been devoted in recent years to the design and synthesize of Schiff bases and their iron(II/III) complexes displaying spin–crossover (SCO) properties with the aim of developing a new generation of memory devices, multifunctional materials, and other magnetic applications (Murray, 2008; Goodwin, 2004 and references therein). The 2–pyridylmethanimine Schiff base derivative has been used as a chelating ligand in mononuclear iron(II) SCO complexes as they often possess field strengths that lie within the right region to facilitate temperature–mediated switching between the high spin (HS) and low spin (LS) states of the iron(II) centers (Guionneau *et al.*, 2001; Capes *et al.*, 2000; Létard *et al.*, 1998; Létard *et al.*, 1997). These ligands are also able to form non–covalent interactions such as hydrogen bonding and weaker electrostatic contracts, which will help to provide diversity to the architectures of structures, and can be associated with subtle effects on the molecular magnetic properties of compounds. For example, the *N*–2–pyridylmethylene–4–(phenylethynyl)aniline (PM–PEA) was employed with [Fe(NCS)_2_] precursor complex to form the mononuclear SCO derivative, [Fe(PM–PEA)_2_(NCS)_2_], exhibiting a thermal hysteresis loop of 37 K (Létard *et al.*, 1997). The crystal structure has been studied at room temperature (HS state) and at 140 K (LS state) suggests that the cooperativity may be attributed to intermolecular π–π stacking between phenyl rings and crucially the strength of the C—H···S interactions. Following the above, a family of iron(II) compounds containing the 2–pyridylmethanimine Schiff base derivatives have been prepared and found to show different SCO behaviors that range from very smooth and incomplete for [Fe(PM–TeA)_2_(NCS)_2_], PM–TeA = *N*–(2'–pyridylmethyl)–4–aminoterphenyl (Guionneau *et al.*, 2001), smooth with almost no hysteresis for [Fe(PM–AzA)_2_(NCS)_2_] and exceptionally abrupt for [Fe(PM–BiA)_2_(NCS)_2_], PM–AzA = *N*–(2'–pyridylmethyl)–4–(phenylazo)aniline, PM–BiA = *N*–(2'–pyridylmethyl)–4–aminobiphenyl (Capes *et al.*, 2000). Our interest in the mononuclear iron(II) SCO complexes has led us to prepare a new *N*–bidentate Schiff base derivative (I), which has the triphenylmethane group attached to the 2–pyridylmethanimine moiety. It is anticipated that weak non–covalent forces such as C—H···π and π–π interactions will help to stabilize the assembly as well as increase the dimensionality of the structure and may also serve as weak exchange pathways between the magnetic metal center. Herein we report the supramolecular structure of (I), which is mediated by both intra– and intermolecular *ef* C—H···π interactions.

The *N*–bidentate Schiff base derivative (I) can be obtained by condensation of 2–pyridinecarboxaldehyde with triphenylmethanamine. Single crystal *X*–ray diffraction analysis reveals that (I) crystallizes in the chiral orthorhombic space group *P*2_1_2_1_2_1_, with two molecules (denoted *A* and *B*) representing the asymmetric unit as shown in Fig. 1. The bond distances within the phenyl (rings 2–4 and 6–8) and pyridine (rings 1 and 5) rings are in the range 1.341 (4) – 1.402 (4) Å, mean 1.381 Å, for molecule *A*, and 1.347 (4) – 1.400 (5) Å, mean 1.381 Å, for molecule *B.* The C—N distances in the imine bonds are 1.268 (4) and 1.273 (4) Å for the C6—N2 and C6'—N2', respectively, which are in agreement with C═N double bond character for the imine group of Schiff bases type (Guionneau *et al.*, 2001). The C7—N2 and the C7'—N2' distances between the imine and the triphenylmethane groups are 1.483 (4) and 1.473 (4) Å, respectively. These are consistent with the values expected for C—N single bonding (Guionneau *et al.*, 2001). The bond angles C5—C6—N2 of 118.5 (2)° and the corresponding C5'—C6'—N2' of 117.9 (2)° are closed to the ideal value of 120°. This fact is further confirmed its *sp*^2^ character.

The molecules *A* and *B* adopt different conformations (the torsion angles C6—N2—C7—C8 = 138.6 (3)° and C6'—N2'—C7'— C8' = -137.1 (2)°) in the crystal of (I) and are linked in inversion–symmetric pairs by *edge-to-face* (*ef*) C—H···π type to form an *A*–*B* dimer. The imine group in both molecules *A* and *B* is approximately coplanar with the pyridine rings, with the N1—C5—C6—N2 and N1'—C5'—C6'—N2' torsion angles of 170.1 (3) and -172.0 (3) Å, respectively. In molecule *A*, the mean plane of the 2–pyridylmethanimine (ring 1—C6═N2) moiety and atom C7 [maximum deviation = 0.101 (2) Å for atom N2] is inclined to the phenyl rings 2, 3, and 4 by 47.2 (1), 84.7 (1), and 59.8 (1)°, respectively. Similarly, the dihedral angles between the mean plane of the corresponding ring 5—C6'═ N2'—C7' [maximum deviation = 0.054 (2) Å for atom C6'] and the phenyl rings 6, 7, and 8 in the molecule *B* are 47.5 (1), 83.9 (1), and 59.4 (1)°, respectively. One of the interesting features of the crystal structure of (I) is the angle between the C═N bond and the rings of the triphenylmethane group. Namely, the angles of N2—C7—C20 (116.3 (2)°) and N2'—C7'—C20' (116.5 (2)°) are much larger than the corresponding angles of N2—C7—C8 (106.4 (2)°), N2'—C7'—C8' (106.2 (2)°), N2—C7—C14 (104.3 (2)°), and N2'—C7'—C14' (104.2 (2)°). This is presumably due to the inversion related intermolecular *ef* C—H···π type in the dimer which lock the molecular conformations.

Several intra– and intermolecular *ef* C—H···π interactions are observed in the crystal structure of (I) and have profound effects on both the molecular and the packing conformations. In the *A*–*B* dimer, hydrogen atoms, H1 and H1' of the pyridine rings (rings 1 and 5) are point toward the centroid (*Cg*) of the phenyl rings (4 and 8) at distance of 2.819 Å (C1—H1···*Cg*8 = 136.9°) for the H1···*Cg*8 and 2.789 Å (C1'—H1'···*Cg*4 = 137.1°) for the H1'···*Cg*1. The 2–pyridylmethanimine moiety of molecules *A* and *B* is almost parallel with the dihedral angle of 0.6 (1)°. It should be noted that the value of C···N contact (C6···N1' = 3.943 (4) Å, C6—H6···N1' = 144.6°; C6'···N1 = 3.929 (4) Å, C6'—H6'···N1 = 144.3°) is greatly longer than the sum of the van der Waals radii [1.70 (C) + 1.54 (N) = 3.24 Å] (Bondi, 1964). Thus, no classical C—H···N hydrogen bonds involving the imine and the pyridine groups are observed in the dimer. There are, however, additional intramolecular C—H···π hydrogen bonding between the adjacent phenyl rings (rings 2–4 and 6–8) with the H···*Cg* distances and the C—H···*Cg* angle in the range 2.897–3.104 Å and 119.8–133.3°, Table 1. As shown in Fig. 2, the dimers are extended into a three–dimensional supramolecular architecture *via* the intermolecular *ef* C—H···π interactions with the H···*Cg* distance and the C—H···*Cg* angle in the range 2.769–3.169 Å and 116.0–145.1°, respectively (Table 1). Finally, no classical hydrogen bonding and π–π stacking between adjacent molecules are observed in the crystal structure of (I). Its packing is mainly based on weak *ef* C—H···π interactions.

### S2. Experimental

To a solution of 2–pyridinecarboxaldehyde (1.90 ml, 0.02 mol) in benzene (100 ml), and a few drops of acetic acid as catalyst was added drop wise with triphenylmethanamine (5.20 g, 0.02 mol) at room temperature. The reaction mixture was stirred under reflux at 110 °C. After 6 h of refluxing, the yellow solution was neutralized with Na_2_CO_3_ (2 mmol), filtered, and concentrated to dryness in *vacuo*. The residue was recrystallized from a mixture of CH_2_Cl_2_ and petroleum ether (2:1, *v*:*v*) to give white crystalline solid of (I). Anal. Found (calcd) for C_25_H_20_N_2_ (348.16): C, 86.15 (86.17); H, 5.81 (5.79); N, 8.05 (8.04).

### S3. Refinement

The C–bound hydrogen atoms were placed in geometrically idealized positions based on chemical coordinations and constrained to ride on their parent atom positions with a C—H distances of 0.95 Å and with *U*_iso_(H) = 1.2*U*_eq_(C) for the aromatic H atoms.

### Crystal data

**Table:**

C_25_H_20_N_2_	*F*(000) = 1472
*M**_r_* = 348.43	*D*_x_ = 1.252 Mg m^−^^3^
Orthorhombic, *P*2_1_2_1_2_1_	Cu *K*α radiation, λ = 1.54178 Å
Hall symbol: P 2ac 2ab	Cell parameters from 3448 reflections
*a* = 7.0719 (5) Å	µ = 0.56 mm^−^^1^
*b* = 16.3972 (8) Å	*T* = 173 K
*c* = 31.880 (2) Å	Block, yellow
*V* = 3696.7 (4) Å^3^	0.30 × 0.26 × 0.22 mm
*Z* = 8	

### Data collection

**Table:**

Agilent Xcalibur (Sapphire3, Gemini ultra) diffractometer	6965 independent reflections
Radiation source: fine-focus sealed tube	5059 reflections with *I* > 2σ(*I*)
Mirror monochromator	*R*_int_ = 0.058
Detector resolution: 8 pixels mm^-1^	θ_max_ = 73.1°, θ_min_ = 9.8°
ω scans	*h* = −8→8
Absorption correction: multi-scan (*CrysAlis PRO* and *CrysAlis RED*; Agilent, 2012)	*k* = −19→15
*T*_min_ = 0.849, *T*_max_ = 0.886	*l* = −28→39
21735 measured reflections	

### Refinement

**Table:**

Refinement on *F*^2^	Secondary atom site location: difference Fourier map
Least-squares matrix: full	Hydrogen site location: inferred from neighbouring sites
*R*[*F*^2^ > 2σ(*F*^2^)] = 0.057	H-atom parameters constrained
*wR*(*F*^2^) = 0.153	*w* = 1/[σ^2^(*F*_o_^2^) + (0.0771*P*)^2^] where *P* = (*F*_o_^2^ + 2*F*_c_^2^)/3
*S* = 1.00	(Δ/σ)_max_ < 0.001
6965 reflections	Δρ_max_ = 0.36 e Å^−^^3^
487 parameters	Δρ_min_ = −0.18 e Å^−^^3^
0 restraints	Absolute structure: Flack (1983), XXX Friedel pairs
Primary atom site location: structure-invariant direct methods	Absolute structure parameter: 0.3 (8)

### Special details

**Table:**

Geometry. All e.s.d.'s (except the e.s.d. in the dihedral angle between two l.s. planes) are estimated using the full covariance matrix. The cell e.s.d.'s are taken into account individually in the estimation of e.s.d.'s in distances, angles and torsion angles; correlations between e.s.d.'s in cell parameters are only used when they are defined by crystal symmetry. An approximate (isotropic) treatment of cell e.s.d.'s is used for estimating e.s.d.'s involving l.s. planes.
Refinement. Refinement of *F*^2^ against ALL reflections. The weighted *R*-factor *wR* and goodness of fit *S* are based on *F*^2^, conventional *R*-factors *R* are based on *F*, with *F* set to zero for negative *F*^2^. The threshold expression of *F*^2^ > σ(*F*^2^) is used only for calculating *R*-factors(gt) *etc*. and is not relevant to the choice of reflections for refinement. *R*-factors based on *F*^2^ are statistically about twice as large as those based on *F*, and *R*- factors based on ALL data will be even larger.

### Fractional atomic coordinates and isotropic or equivalent isotropic displacement parameters (Å^2^)

**Table:**

	*x*	*y*	*z*	*U*_iso_*/*U*_eq_	
N1	0.3234 (4)	0.72675 (16)	0.72533 (8)	0.0395 (6)	
N2	0.0763 (4)	0.67303 (16)	0.63297 (8)	0.0368 (6)	
C1	0.4649 (5)	0.7751 (2)	0.73827 (11)	0.0408 (7)	
H1	0.4933	0.7761	0.7674	0.049*	
C2	0.5717 (5)	0.8232 (2)	0.71190 (11)	0.0419 (8)	
H2	0.6713	0.8561	0.7226	0.050*	
C3	0.5300 (5)	0.8225 (2)	0.66932 (10)	0.0419 (8)	
H3	0.5988	0.8559	0.6504	0.050*	
C4	0.3861 (5)	0.77209 (19)	0.65489 (10)	0.0385 (7)	
H4	0.3559	0.7696	0.6259	0.046*	
C5	0.2876 (4)	0.72545 (18)	0.68395 (9)	0.0339 (7)	
C6	0.1354 (5)	0.66888 (19)	0.67048 (10)	0.0359 (7)	
H6	0.0833	0.6304	0.6895	0.043*	
C7	−0.0641 (4)	0.61515 (18)	0.61548 (9)	0.0335 (7)	
C8	−0.2019 (5)	0.66622 (19)	0.58873 (9)	0.0355 (7)	
C13	−0.1588 (5)	0.7448 (2)	0.57610 (11)	0.0465 (8)	
H13	−0.0432	0.7689	0.5848	0.056*	
C12	−0.2826 (6)	0.7891 (2)	0.55077 (12)	0.0560 (10)	
H12	−0.2515	0.8432	0.5425	0.067*	
C11	−0.4510 (6)	0.7541 (2)	0.53771 (11)	0.0510 (9)	
H11	−0.5344	0.7835	0.5199	0.061*	
C10	−0.4964 (5)	0.6768 (2)	0.55064 (11)	0.0470 (9)	
H10	−0.6125	0.6529	0.5421	0.056*	
C9	−0.3737 (5)	0.6334 (2)	0.57618 (11)	0.0432 (8)	
H9	−0.4079	0.5802	0.5852	0.052*	
C14	0.0542 (4)	0.55700 (18)	0.58757 (9)	0.0338 (7)	
C15	0.0086 (5)	0.5395 (2)	0.54651 (10)	0.0426 (8)	
H15	−0.0942	0.5670	0.5335	0.051*	
C16	0.1108 (6)	0.4824 (2)	0.52396 (11)	0.0491 (9)	
H16	0.0777	0.4712	0.4957	0.059*	
C17	0.2588 (5)	0.4419 (2)	0.54197 (11)	0.0475 (9)	
H17	0.3262	0.4019	0.5265	0.057*	
C18	0.3102 (5)	0.4593 (2)	0.58270 (10)	0.0421 (8)	
H18	0.4148	0.4322	0.5952	0.051*	
C19	0.2088 (5)	0.51641 (19)	0.60531 (10)	0.0390 (7)	
H19	0.2447	0.5283	0.6334	0.047*	
C20	−0.1792 (4)	0.56558 (19)	0.64745 (9)	0.0330 (6)	
C21	−0.2010 (5)	0.48179 (19)	0.64471 (10)	0.0373 (7)	
H21	−0.1317	0.4526	0.6241	0.045*	
C22	−0.3221 (5)	0.4391 (2)	0.67150 (10)	0.0403 (7)	
H22	−0.3347	0.3817	0.6688	0.048*	
C23	−0.4230 (5)	0.4802 (2)	0.70172 (10)	0.0407 (7)	
H23	−0.5065	0.4516	0.7198	0.049*	
C24	−0.4017 (5)	0.5638 (2)	0.70542 (10)	0.0418 (8)	
H24	−0.4700	0.5925	0.7264	0.050*	
C25	−0.2821 (5)	0.60567 (19)	0.67889 (10)	0.0374 (7)	
H25	−0.2691	0.6630	0.6820	0.045*	
N1'	0.1678 (4)	0.52217 (16)	0.76822 (8)	0.0386 (6)	
C1'	0.0245 (5)	0.4739 (2)	0.75559 (11)	0.0412 (8)	
H1'	−0.0053	0.4722	0.7266	0.049*	
N2'	0.4146 (4)	0.57659 (16)	0.86033 (8)	0.0363 (6)	
C2'	−0.0814 (5)	0.4267 (2)	0.78293 (11)	0.0447 (8)	
H2'	−0.1825	0.3941	0.7728	0.054*	
C3'	−0.0381 (5)	0.4277 (2)	0.82482 (11)	0.0453 (8)	
H3'	−0.1075	0.3952	0.8441	0.054*	
C4'	0.1078 (5)	0.4768 (2)	0.83851 (10)	0.0389 (7)	
H4'	0.1404	0.4789	0.8674	0.047*	
C5'	0.2058 (4)	0.52291 (18)	0.80935 (9)	0.0335 (6)	
C6'	0.3573 (4)	0.58008 (19)	0.82251 (9)	0.0348 (7)	
H6'	0.4100	0.6181	0.8033	0.042*	
C7'	0.5550 (4)	0.63331 (18)	0.87805 (9)	0.0315 (6)	
C8'	0.6910 (5)	0.58111 (19)	0.90443 (9)	0.0333 (6)	
C9'	0.8659 (5)	0.6119 (2)	0.91612 (10)	0.0400 (7)	
H9'	0.9025	0.6647	0.9069	0.048*	
C10'	0.9893 (5)	0.5664 (2)	0.94125 (10)	0.0448 (8)	
H10'	1.1085	0.5883	0.9490	0.054*	
C11'	0.9380 (6)	0.4904 (2)	0.95454 (11)	0.0506 (9)	
H11'	1.0206	0.4597	0.9720	0.061*	
C12'	0.7658 (6)	0.4580 (2)	0.94262 (12)	0.0507 (9)	
H12'	0.7315	0.4047	0.9515	0.061*	
C13'	0.6430 (5)	0.5026 (2)	0.91780 (10)	0.0438 (8)	
H13'	0.5251	0.4797	0.9098	0.053*	
C14'	0.4373 (4)	0.69107 (18)	0.90604 (9)	0.0330 (7)	
C15'	0.4871 (5)	0.7085 (2)	0.94706 (10)	0.0403 (8)	
H15'	0.5904	0.6807	0.9596	0.048*	
C16'	0.3873 (6)	0.7665 (2)	0.97014 (11)	0.0488 (9)	
H16'	0.4250	0.7788	0.9980	0.059*	
C17'	0.2338 (5)	0.8060 (2)	0.95266 (10)	0.0444 (8)	
H17'	0.1664	0.8458	0.9683	0.053*	
C18'	0.1797 (5)	0.7872 (2)	0.91230 (10)	0.0409 (8)	
H18'	0.0728	0.8133	0.9003	0.049*	
C19'	0.2796 (4)	0.73047 (19)	0.88923 (10)	0.0375 (7)	
H19'	0.2404	0.7181	0.8615	0.045*	
C20'	0.6711 (4)	0.68358 (18)	0.84642 (9)	0.0308 (6)	
C21'	0.6930 (4)	0.76739 (19)	0.84992 (9)	0.0348 (7)	
H21'	0.6231	0.7962	0.8706	0.042*	
C22'	0.8149 (5)	0.8098 (2)	0.82383 (10)	0.0392 (7)	
H22'	0.8284	0.8672	0.8268	0.047*	
C23'	0.9176 (5)	0.7686 (2)	0.79330 (10)	0.0423 (8)	
H23'	1.0034	0.7973	0.7758	0.051*	
C24'	0.8937 (5)	0.68586 (19)	0.78877 (10)	0.0388 (7)	
H24'	0.9604	0.6575	0.7674	0.047*	
C25'	0.7734 (5)	0.64361 (19)	0.81508 (9)	0.0361 (7)	
H25'	0.7599	0.5863	0.8118	0.043*	

### Atomic displacement parameters (Å^2^)

**Table:**

	*U*^11^	*U*^22^	*U*^33^	*U*^12^	*U*^13^	*U*^23^
N1	0.0380 (14)	0.0299 (15)	0.0505 (15)	−0.0005 (12)	−0.0043 (12)	−0.0053 (12)
N2	0.0331 (14)	0.0311 (14)	0.0461 (14)	−0.0026 (11)	−0.0023 (11)	−0.0050 (11)
C1	0.0409 (17)	0.0314 (19)	0.0502 (18)	0.0022 (15)	−0.0073 (15)	−0.0054 (15)
C2	0.0355 (17)	0.0300 (18)	0.060 (2)	−0.0027 (13)	−0.0094 (15)	−0.0092 (15)
C3	0.0403 (18)	0.0332 (18)	0.0521 (18)	−0.0052 (14)	−0.0007 (15)	−0.0021 (14)
C4	0.0396 (17)	0.0265 (17)	0.0495 (17)	0.0003 (14)	0.0015 (14)	−0.0030 (14)
C5	0.0333 (15)	0.0263 (16)	0.0420 (16)	0.0035 (12)	−0.0037 (13)	−0.0055 (13)
C6	0.0359 (16)	0.0253 (17)	0.0465 (17)	−0.0004 (13)	0.0014 (13)	−0.0039 (13)
C7	0.0322 (14)	0.0267 (16)	0.0415 (16)	−0.0058 (12)	−0.0007 (13)	−0.0021 (12)
C8	0.0372 (16)	0.0290 (17)	0.0401 (15)	−0.0018 (13)	0.0027 (13)	−0.0010 (13)
C13	0.049 (2)	0.037 (2)	0.0540 (19)	−0.0083 (16)	−0.0019 (16)	0.0041 (15)
C12	0.065 (3)	0.045 (2)	0.059 (2)	0.002 (2)	−0.006 (2)	0.0096 (17)
C11	0.056 (2)	0.043 (2)	0.053 (2)	0.0137 (17)	−0.0087 (17)	−0.0002 (16)
C10	0.0377 (18)	0.050 (2)	0.0531 (19)	0.0015 (16)	−0.0034 (15)	0.0010 (16)
C9	0.0402 (18)	0.0307 (18)	0.059 (2)	−0.0017 (14)	−0.0037 (15)	0.0065 (15)
C14	0.0317 (15)	0.0266 (16)	0.0431 (16)	−0.0042 (12)	0.0025 (13)	0.0002 (13)
C15	0.0359 (17)	0.047 (2)	0.0454 (18)	0.0007 (16)	−0.0005 (14)	−0.0005 (15)
C16	0.050 (2)	0.049 (2)	0.0481 (18)	0.0021 (18)	0.0021 (16)	−0.0076 (16)
C17	0.047 (2)	0.043 (2)	0.053 (2)	0.0017 (17)	0.0104 (16)	−0.0014 (16)
C18	0.0352 (17)	0.0341 (19)	0.0571 (19)	−0.0004 (14)	0.0041 (15)	0.0052 (14)
C19	0.0350 (16)	0.0318 (18)	0.0502 (17)	−0.0028 (13)	−0.0005 (14)	0.0003 (14)
C20	0.0318 (15)	0.0264 (16)	0.0408 (15)	0.0008 (12)	−0.0018 (12)	−0.0020 (12)
C21	0.0386 (17)	0.0269 (17)	0.0464 (17)	−0.0060 (14)	0.0037 (14)	−0.0038 (13)
C22	0.0430 (18)	0.0280 (17)	0.0499 (17)	−0.0053 (14)	0.0003 (15)	0.0005 (13)
C23	0.0346 (16)	0.040 (2)	0.0474 (17)	−0.0012 (14)	0.0032 (14)	0.0097 (14)
C24	0.0387 (17)	0.040 (2)	0.0473 (18)	0.0079 (15)	0.0038 (14)	0.0039 (14)
C25	0.0379 (16)	0.0262 (17)	0.0481 (17)	0.0054 (13)	0.0046 (14)	0.0021 (13)
N1'	0.0375 (14)	0.0302 (15)	0.0480 (14)	0.0020 (11)	−0.0039 (11)	−0.0060 (12)
C1'	0.0427 (17)	0.0289 (19)	0.0519 (18)	0.0064 (14)	−0.0076 (14)	−0.0104 (14)
N2'	0.0331 (13)	0.0301 (14)	0.0456 (14)	−0.0055 (11)	−0.0010 (11)	−0.0025 (11)
C2'	0.0357 (17)	0.0340 (18)	0.064 (2)	−0.0061 (14)	−0.0058 (16)	−0.0139 (16)
C3'	0.0401 (18)	0.0351 (19)	0.061 (2)	−0.0049 (14)	0.0048 (16)	−0.0064 (16)
C4'	0.0385 (17)	0.0319 (18)	0.0462 (17)	−0.0006 (14)	0.0031 (14)	−0.0048 (14)
C5'	0.0335 (15)	0.0243 (16)	0.0428 (15)	0.0037 (12)	−0.0029 (13)	−0.0049 (12)
C6'	0.0370 (16)	0.0239 (17)	0.0435 (17)	−0.0012 (12)	−0.0038 (13)	−0.0021 (13)
C7'	0.0290 (14)	0.0265 (16)	0.0388 (15)	−0.0028 (12)	−0.0010 (12)	−0.0025 (12)
C8'	0.0365 (15)	0.0267 (16)	0.0368 (15)	0.0007 (13)	0.0003 (12)	−0.0002 (12)
C9'	0.0385 (17)	0.0355 (19)	0.0459 (18)	−0.0047 (14)	−0.0047 (14)	0.0032 (14)
C10'	0.0358 (17)	0.050 (2)	0.0484 (18)	0.0008 (16)	−0.0064 (14)	0.0027 (16)
C11'	0.051 (2)	0.050 (2)	0.0513 (19)	0.0112 (18)	−0.0074 (17)	0.0075 (17)
C12'	0.060 (2)	0.030 (2)	0.063 (2)	0.0001 (17)	−0.0034 (19)	0.0102 (15)
C13'	0.046 (2)	0.0341 (19)	0.0510 (18)	−0.0077 (15)	−0.0027 (15)	0.0047 (14)
C14'	0.0325 (15)	0.0267 (16)	0.0398 (15)	−0.0073 (12)	0.0017 (12)	0.0016 (12)
C15'	0.0358 (17)	0.045 (2)	0.0399 (16)	0.0008 (15)	−0.0025 (13)	0.0014 (14)
C16'	0.050 (2)	0.047 (2)	0.0494 (19)	0.0037 (18)	0.0028 (16)	−0.0078 (16)
C17'	0.0445 (19)	0.038 (2)	0.0506 (19)	0.0042 (16)	0.0092 (15)	0.0007 (15)
C18'	0.0340 (16)	0.035 (2)	0.0533 (19)	0.0007 (14)	0.0028 (14)	0.0058 (14)
C19'	0.0332 (15)	0.0323 (18)	0.0469 (17)	−0.0017 (13)	−0.0008 (13)	0.0043 (14)
C20'	0.0259 (13)	0.0249 (16)	0.0416 (15)	−0.0033 (11)	−0.0026 (12)	−0.0005 (12)
C21'	0.0344 (16)	0.0277 (17)	0.0422 (16)	−0.0030 (13)	0.0005 (13)	−0.0034 (13)
C22'	0.0396 (17)	0.0289 (18)	0.0492 (17)	−0.0049 (14)	−0.0004 (15)	0.0030 (13)
C23'	0.0366 (17)	0.043 (2)	0.0472 (18)	0.0011 (15)	0.0050 (14)	0.0106 (15)
C24'	0.0386 (17)	0.0349 (19)	0.0430 (17)	0.0078 (14)	0.0067 (14)	0.0047 (14)
C25'	0.0387 (16)	0.0256 (16)	0.0440 (16)	0.0019 (13)	0.0019 (13)	−0.0004 (13)

### Geometric parameters (Å, º)

**Table:**

N1—C1	1.341 (4)	N1'—C5'	1.339 (4)
N1—C5	1.344 (4)	N1'—C1'	1.347 (4)
N2—C6	1.268 (4)	C1'—C2'	1.385 (5)
N2—C7	1.483 (4)	C1'—H1'	0.9500
C1—C2	1.379 (5)	N2'—C6'	1.273 (4)
C1—H1	0.9500	N2'—C7'	1.473 (4)
C2—C3	1.389 (5)	C2'—C3'	1.370 (5)
C2—H2	0.9500	C2'—H2'	0.9500
C3—C4	1.389 (5)	C3'—C4'	1.379 (5)
C3—H3	0.9500	C3'—H3'	0.9500
C4—C5	1.388 (4)	C4'—C5'	1.384 (4)
C4—H4	0.9500	C4'—H4'	0.9500
C5—C6	1.485 (4)	C5'—C6'	1.484 (4)
C6—H6	0.9500	C6'—H6'	0.9500
C7—C20	1.537 (4)	C7'—C8'	1.538 (4)
C7—C8	1.542 (4)	C7'—C20'	1.539 (4)
C7—C14	1.550 (4)	C7'—C14'	1.545 (4)
C8—C13	1.384 (4)	C8'—C9'	1.387 (4)
C8—C9	1.388 (5)	C8'—C13'	1.397 (4)
C13—C12	1.395 (5)	C9'—C10'	1.400 (5)
C13—H13	0.9500	C9'—H9'	0.9500
C12—C11	1.386 (6)	C10'—C11'	1.366 (5)
C12—H12	0.9500	C10'—H10'	0.9500
C11—C10	1.371 (5)	C11'—C12'	1.381 (6)
C11—H11	0.9500	C11'—H11'	0.9500
C10—C9	1.387 (5)	C12'—C13'	1.384 (5)
C10—H10	0.9500	C12'—H12'	0.9500
C9—H9	0.9500	C13'—H13'	0.9500
C14—C15	1.378 (4)	C14'—C15'	1.384 (4)
C14—C19	1.399 (4)	C14'—C19'	1.396 (4)
C15—C16	1.385 (5)	C15'—C16'	1.393 (5)
C15—H15	0.9500	C15'—H15'	0.9500
C16—C17	1.366 (5)	C16'—C17'	1.382 (5)
C16—H16	0.9500	C16'—H16'	0.9500
C17—C18	1.378 (5)	C17'—C18'	1.378 (5)
C17—H17	0.9500	C17'—H17'	0.9500
C18—C19	1.383 (4)	C18'—C19'	1.380 (4)
C18—H18	0.9500	C18'—H18'	0.9500
C19—H19	0.9500	C19'—H19'	0.9500
C20—C21	1.385 (4)	C20'—C21'	1.388 (4)
C20—C25	1.402 (4)	C20'—C25'	1.397 (4)
C21—C22	1.397 (4)	C21'—C22'	1.385 (4)
C21—H21	0.9500	C21'—H21'	0.9500
C22—C23	1.375 (5)	C22'—C23'	1.390 (5)
C22—H22	0.9500	C22'—H22'	0.9500
C23—C24	1.383 (5)	C23'—C24'	1.375 (5)
C23—H23	0.9500	C23'—H23'	0.9500
C24—C25	1.379 (5)	C24'—C25'	1.381 (4)
C24—H24	0.9500	C24'—H24'	0.9500
C25—H25	0.9500	C25'—H25'	0.9500
			
C1—N1—C5	116.9 (3)	C5'—N1'—C1'	116.7 (3)
C6—N2—C7	122.8 (3)	N1'—C1'—C2'	123.2 (3)
N1—C1—C2	124.0 (3)	N1'—C1'—H1'	118.4
N1—C1—H1	118.0	C2'—C1'—H1'	118.4
C2—C1—H1	118.0	C6'—N2'—C7'	123.3 (3)
C1—C2—C3	118.4 (3)	C3'—C2'—C1'	119.1 (3)
C1—C2—H2	120.8	C3'—C2'—H2'	120.5
C3—C2—H2	120.8	C1'—C2'—H2'	120.5
C2—C3—C4	119.0 (3)	C2'—C3'—C4'	118.8 (3)
C2—C3—H3	120.5	C2'—C3'—H3'	120.6
C4—C3—H3	120.5	C4'—C3'—H3'	120.6
C5—C4—C3	118.3 (3)	C3'—C4'—C5'	118.7 (3)
C5—C4—H4	120.8	C3'—C4'—H4'	120.6
C3—C4—H4	120.8	C5'—C4'—H4'	120.6
N1—C5—C4	123.5 (3)	N1'—C5'—C4'	123.5 (3)
N1—C5—C6	115.5 (3)	N1'—C5'—C6'	115.3 (3)
C4—C5—C6	121.0 (3)	C4'—C5'—C6'	121.1 (3)
N2—C6—C5	118.6 (3)	N2'—C6'—C5'	118.0 (3)
N2—C6—H6	120.7	N2'—C6'—H6'	121.0
C5—C6—H6	120.7	C5'—C6'—H6'	121.0
N2—C7—C20	116.3 (2)	N2'—C7'—C8'	106.2 (2)
N2—C7—C8	106.5 (2)	N2'—C7'—C20'	116.5 (2)
C20—C7—C8	108.6 (3)	C8'—C7'—C20'	108.8 (2)
N2—C7—C14	104.4 (2)	N2'—C7'—C14'	104.2 (2)
C20—C7—C14	110.0 (3)	C8'—C7'—C14'	111.2 (2)
C8—C7—C14	111.0 (2)	C20'—C7'—C14'	109.7 (2)
C13—C8—C9	118.0 (3)	C9'—C8'—C13'	118.0 (3)
C13—C8—C7	121.9 (3)	C9'—C8'—C7'	120.1 (3)
C9—C8—C7	120.1 (3)	C13'—C8'—C7'	121.9 (3)
C8—C13—C12	121.0 (4)	C8'—C9'—C10'	121.0 (3)
C8—C13—H13	119.5	C8'—C9'—H9'	119.5
C12—C13—H13	119.5	C10'—C9'—H9'	119.5
C11—C12—C13	119.8 (4)	C11'—C10'—C9'	119.9 (3)
C11—C12—H12	120.1	C11'—C10'—H10'	120.1
C13—C12—H12	120.1	C9'—C10'—H10'	120.1
C10—C11—C12	119.6 (4)	C10'—C11'—C12'	120.0 (3)
C10—C11—H11	120.2	C10'—C11'—H11'	120.0
C12—C11—H11	120.2	C12'—C11'—H11'	120.0
C11—C10—C9	120.3 (4)	C11'—C12'—C13'	120.5 (4)
C11—C10—H10	119.8	C11'—C12'—H12'	119.8
C9—C10—H10	119.8	C13'—C12'—H12'	119.8
C10—C9—C8	121.2 (3)	C12'—C13'—C8'	120.6 (3)
C10—C9—H9	119.4	C12'—C13'—H13'	119.7
C8—C9—H9	119.4	C8'—C13'—H13'	119.7
C15—C14—C19	117.9 (3)	C15'—C14'—C19'	118.0 (3)
C15—C14—C7	123.1 (3)	C15'—C14'—C7'	122.4 (3)
C19—C14—C7	118.9 (3)	C19'—C14'—C7'	119.5 (3)
C14—C15—C16	120.8 (3)	C14'—C15'—C16'	120.7 (3)
C14—C15—H15	119.6	C14'—C15'—H15'	119.6
C16—C15—H15	119.6	C16'—C15'—H15'	119.6
C17—C16—C15	120.7 (3)	C17'—C16'—C15'	120.3 (3)
C17—C16—H16	119.7	C17'—C16'—H16'	119.8
C15—C16—H16	119.7	C15'—C16'—H16'	119.8
C16—C17—C18	119.9 (3)	C18'—C17'—C16'	119.3 (3)
C16—C17—H17	120.1	C18'—C17'—H17'	120.3
C18—C17—H17	120.1	C16'—C17'—H17'	120.3
C17—C18—C19	119.6 (3)	C17'—C18'—C19'	120.4 (3)
C17—C18—H18	120.2	C17'—C18'—H18'	119.8
C19—C18—H18	120.2	C19'—C18'—H18'	119.8
C18—C19—C14	121.1 (3)	C18'—C19'—C14'	121.1 (3)
C18—C19—H19	119.4	C18'—C19'—H19'	119.5
C14—C19—H19	119.4	C14'—C19'—H19'	119.5
C21—C20—C25	116.9 (3)	C21'—C20'—C25'	117.7 (3)
C21—C20—C7	122.8 (3)	C21'—C20'—C7'	122.5 (3)
C25—C20—C7	120.1 (3)	C25'—C20'—C7'	119.6 (3)
C20—C21—C22	121.7 (3)	C22'—C21'—C20'	121.3 (3)
C20—C21—H21	119.1	C22'—C21'—H21'	119.4
C22—C21—H21	119.1	C20'—C21'—H21'	119.4
C23—C22—C21	120.1 (3)	C21'—C22'—C23'	120.1 (3)
C23—C22—H22	120.0	C21'—C22'—H22'	120.0
C21—C22—H22	120.0	C23'—C22'—H22'	120.0
C22—C23—C24	119.2 (3)	C24'—C23'—C22'	119.3 (3)
C22—C23—H23	120.4	C24'—C23'—H23'	120.4
C24—C23—H23	120.4	C22'—C23'—H23'	120.4
C25—C24—C23	120.5 (3)	C23'—C24'—C25'	120.4 (3)
C25—C24—H24	119.7	C23'—C24'—H24'	119.8
C23—C24—H24	119.7	C25'—C24'—H24'	119.8
C24—C25—C20	121.5 (3)	C24'—C25'—C20'	121.2 (3)
C24—C25—H25	119.2	C24'—C25'—H25'	119.4
C20—C25—H25	119.2	C20'—C25'—H25'	119.4
			
C5—N1—C1—C2	0.9 (5)	C5'—N1'—C1'—C2'	0.1 (5)
N1—C1—C2—C3	0.5 (5)	N1'—C1'—C2'—C3'	−0.9 (5)
C1—C2—C3—C4	−1.5 (5)	C1'—C2'—C3'—C4'	1.0 (5)
C2—C3—C4—C5	1.1 (5)	C2'—C3'—C4'—C5'	−0.3 (5)
C1—N1—C5—C4	−1.3 (5)	C1'—N1'—C5'—C4'	0.7 (4)
C1—N1—C5—C6	177.6 (3)	C1'—N1'—C5'—C6'	−176.8 (3)
C3—C4—C5—N1	0.3 (5)	C3'—C4'—C5'—N1'	−0.6 (5)
C3—C4—C5—C6	−178.5 (3)	C3'—C4'—C5'—C6'	176.7 (3)
C7—N2—C6—C5	175.0 (3)	C7'—N2'—C6'—C5'	−175.9 (3)
N1—C5—C6—N2	170.1 (3)	N1'—C5'—C6'—N2'	−172.0 (3)
C4—C5—C6—N2	−10.9 (5)	C4'—C5'—C6'—N2'	10.5 (4)
C6—N2—C7—C20	17.4 (4)	C6'—N2'—C7'—C8'	−137.2 (3)
C6—N2—C7—C8	138.6 (3)	C6'—N2'—C7'—C20'	−15.8 (4)
C6—N2—C7—C14	−103.9 (3)	C6'—N2'—C7'—C14'	105.2 (3)
N2—C7—C8—C13	15.9 (4)	N2'—C7'—C8'—C9'	162.9 (3)
C20—C7—C8—C13	141.9 (3)	C20'—C7'—C8'—C9'	36.7 (4)
C14—C7—C8—C13	−97.1 (3)	C14'—C7'—C8'—C9'	−84.3 (3)
N2—C7—C8—C9	−164.9 (3)	N2'—C7'—C8'—C13'	−18.0 (4)
C20—C7—C8—C9	−38.9 (4)	C20'—C7'—C8'—C13'	−144.2 (3)
C14—C7—C8—C9	82.1 (4)	C14'—C7'—C8'—C13'	94.8 (3)
C9—C8—C13—C12	−1.3 (5)	C13'—C8'—C9'—C10'	−1.2 (5)
C7—C8—C13—C12	177.9 (3)	C7'—C8'—C9'—C10'	178.0 (3)
C8—C13—C12—C11	−0.4 (6)	C8'—C9'—C10'—C11'	0.1 (5)
C13—C12—C11—C10	1.5 (6)	C9'—C10'—C11'—C12'	1.1 (6)
C12—C11—C10—C9	−0.9 (6)	C10'—C11'—C12'—C13'	−1.1 (6)
C11—C10—C9—C8	−0.9 (5)	C11'—C12'—C13'—C8'	−0.1 (6)
C13—C8—C9—C10	2.0 (5)	C9'—C8'—C13'—C12'	1.2 (5)
C7—C8—C9—C10	−177.2 (3)	C7'—C8'—C13'—C12'	−178.0 (3)
N2—C7—C14—C15	−129.1 (3)	N2'—C7'—C14'—C15'	130.6 (3)
C20—C7—C14—C15	105.4 (3)	C8'—C7'—C14'—C15'	16.5 (4)
C8—C7—C14—C15	−14.8 (4)	C20'—C7'—C14'—C15'	−103.9 (3)
N2—C7—C14—C19	54.7 (4)	N2'—C7'—C14'—C19'	−52.4 (3)
C20—C7—C14—C19	−70.8 (3)	C8'—C7'—C14'—C19'	−166.5 (3)
C8—C7—C14—C19	169.0 (3)	C20'—C7'—C14'—C19'	73.0 (3)
C19—C14—C15—C16	1.2 (5)	C19'—C14'—C15'—C16'	−2.8 (5)
C7—C14—C15—C16	−175.0 (3)	C7'—C14'—C15'—C16'	174.2 (3)
C14—C15—C16—C17	0.2 (6)	C14'—C15'—C16'—C17'	1.6 (6)
C15—C16—C17—C18	−1.5 (6)	C15'—C16'—C17'—C18'	0.5 (6)
C16—C17—C18—C19	1.4 (5)	C16'—C17'—C18'—C19'	−1.2 (5)
C17—C18—C19—C14	0.1 (5)	C17'—C18'—C19'—C14'	−0.1 (5)
C15—C14—C19—C18	−1.4 (5)	C15'—C14'—C19'—C18'	2.1 (5)
C7—C14—C19—C18	175.1 (3)	C7'—C14'—C19'—C18'	−175.0 (3)
N2—C7—C20—C21	−130.8 (3)	N2'—C7'—C20'—C21'	130.7 (3)
C8—C7—C20—C21	109.1 (3)	C8'—C7'—C20'—C21'	−109.3 (3)
C14—C7—C20—C21	−12.5 (4)	C14'—C7'—C20'—C21'	12.6 (4)
N2—C7—C20—C25	55.1 (4)	N2'—C7'—C20'—C25'	−55.4 (4)
C8—C7—C20—C25	−64.9 (4)	C8'—C7'—C20'—C25'	64.6 (3)
C14—C7—C20—C25	173.4 (3)	C14'—C7'—C20'—C25'	−173.5 (3)
C25—C20—C21—C22	1.1 (5)	C25'—C20'—C21'—C22'	−1.3 (5)
C7—C20—C21—C22	−173.2 (3)	C7'—C20'—C21'—C22'	172.7 (3)
C20—C21—C22—C23	−0.3 (5)	C20'—C21'—C22'—C23'	0.3 (5)
C21—C22—C23—C24	−0.6 (5)	C21'—C22'—C23'—C24'	1.4 (5)
C22—C23—C24—C25	0.6 (5)	C22'—C23'—C24'—C25'	−2.0 (5)
C23—C24—C25—C20	0.2 (5)	C23'—C24'—C25'—C20'	0.9 (5)
C21—C20—C25—C24	−1.0 (5)	C21'—C20'—C25'—C24'	0.7 (5)
C7—C20—C25—C24	173.4 (3)	C7'—C20'—C25'—C24'	−173.5 (3)
