# Supplementary figures and images for: Crystal structure of (pyridin-2-yl­methyl­idene)(tri­phenyl­meth­yl)amine
Source: Acta Crystallogr Sect E Struct Rep Online. 2014 Sep 6;70(Pt 10):o1094–5. doi: 10.1107/S160053681401959X (PMC4257176; doi:10.1107/S160053681401959X)

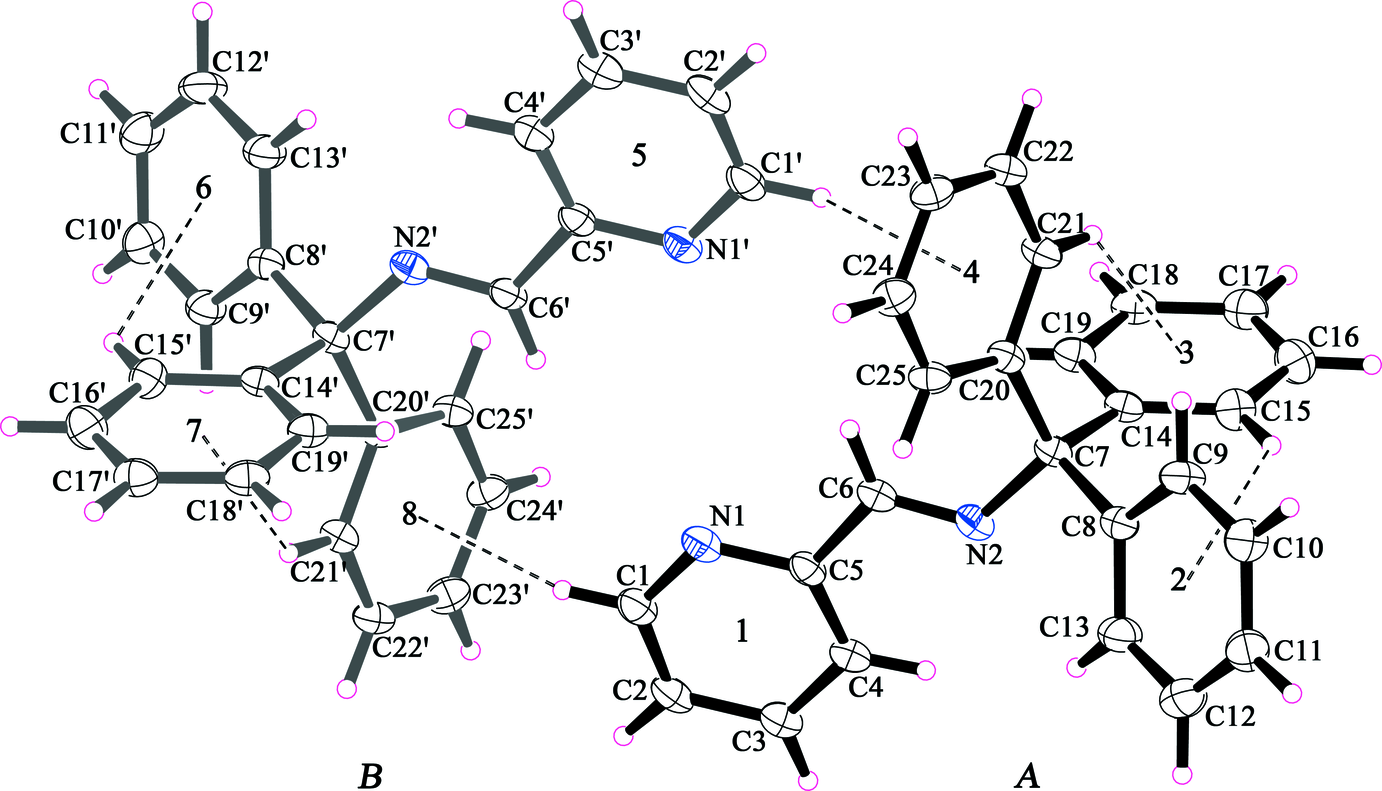

Supplement: Supplementary file 5 [file e-70-o1094-fig1.tif]

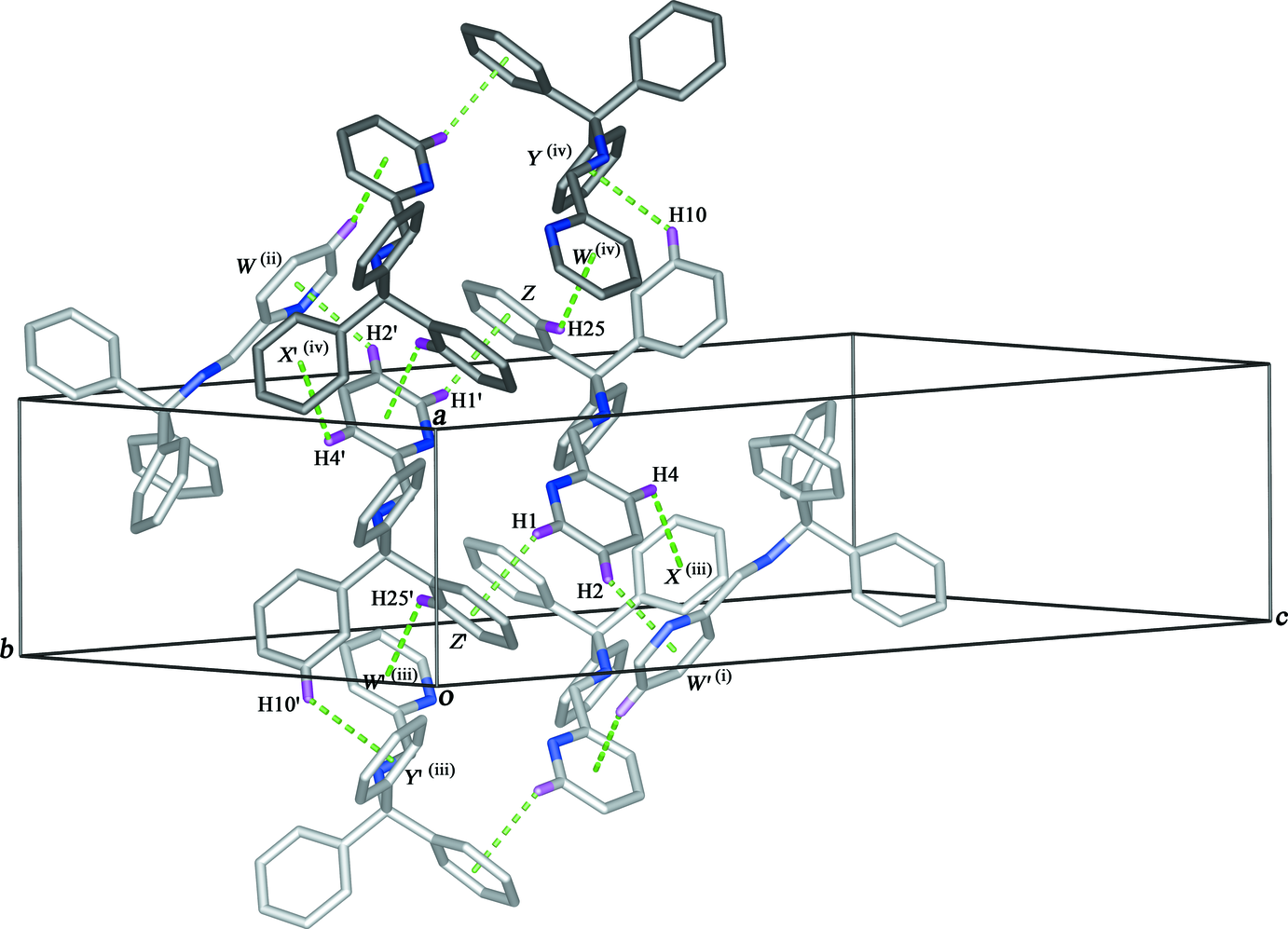

Supplement: Supplementary file 6 [file e-70-o1094-fig2.tif]
